# Feasibility of predicting free-breathing body contours from biplanar CT scout images for surface-guided DIBH radiotherapy

**DOI:** 10.1016/j.phro.2026.101023

**Published:** 2026-06-17

**Authors:** Yoonsuk Huh, Seonghee Kang, Jaewon Yang, Jung-in Kim

**Affiliations:** a Department of Radiation Oncology, Seoul National University Hospital, Seoul, Republic of Korea; b Institute of Radiation Medicine, Seoul National University Medical Research Center, Seoul, Republic of Korea; c Biomedical Research Institute, Seoul National University Hospital, Seoul, Republic of Korea; d Department of Radiation Oncology, Seoul National University College of Medicine, Seoul, Republic of Korea; e Biomedical Research Institute, Department of Radiology, UT Southwestern Medical Center, TX, United States

**Keywords:** CT scout images, Deep-learning, Body contouring, Surface guided radiation therapy (SGRT), Deep inspiration breath hold (DIBH)

## Abstract

**Background and purpose:**

Surface-guided deep inspiration breath-hold radiotherapy uses deep inspiration breath-hold CT for treatment planning, whereas a free-breathing body contour is needed for baseline surface registration. This requires an additional free-breathing CT acquisition. This study investigated the feasibility of generating a three-dimensional free-breathing body contour directly from routine biplanar CT scout images using deep learning.

**Material and methods:**

A total of 173 thoracic CT-simulation studies with paired coronal and sagittal CT scout images and corresponding free-breathing CT images were retrospectively collected. The CT-derived body mask was generated using an automated threshold- and morphology-based procedure and used as the reference contour. The dataset was divided into training, validation, and test cohorts of 121, 26, and 26 studies, respectively. Geometric performance was evaluated using the Dice coefficient, 95th percentile Hausdorff distance, and mean surface distance.

**Results:**

In the test cohort, the predicted contours demonstrated high geometric agreement with the reference contours, with a Dice coefficient of 0.979 ± 0.017, 95th percentile Hausdorff distance of 4.23 ± 2.87 mm, and mean surface distance of 1.05 ± 0.73 mm. Slice-wise analysis showed similar performance. Larger and more symmetric, geometrically regular torso shapes were associated with better performance.

**Conclusions:**

A three-dimensional free-breathing body contour was generated from routine biplanar CT scout images with high geometric agreement to the CT-derived reference contour. This approach may reduce the need for additional free-breathing CT acquisition for patient setup, but prospective validation in clinical setup, registration, and gating workflows is required.

## Highlights

• Deep learning generated body contours from CT scout images.
• Test-set predictions closely matched reference contours.
• Slice-wise analysis showed consistent axial agreement.
• Regular body shapes were associated with higher accuracy.
• The method may reduce additional free-breathing CT acquisition.

## Graphical abstract

**Figure:**
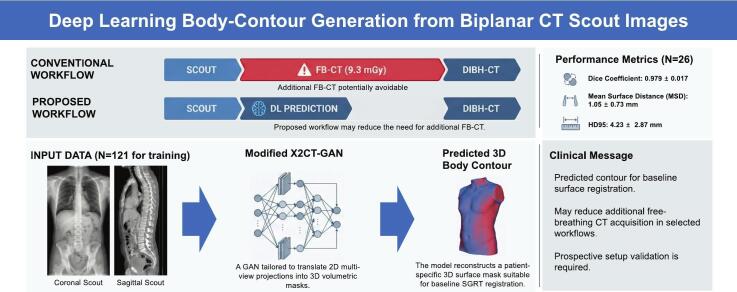

## 1 Introduction

In modern radiation therapy, accurate patient positioning is critical for precise dose delivery and minimizing exposure to normal tissues [1], [2], [3]. Traditionally, visible skin markers have been used for alignment, but they introduce limitations including patient discomfort, hygiene concerns, and an increased risk of setup error from motion or marker fading [4], [5]. Surface-guided radiation therapy (SGRT) has therefore emerged as a marker-less solution that uses real-time 3D surface imaging to monitor patient position throughout simulation and treatment [6], [7], [8]. Deep-inspiration breath-hold (DIBH) has been shown to reduce cardiac and pulmonary doses in breast and thoracic radiotherapy while maintaining target coverage. SGRT provides a marker-less approach for patient setup, surface monitoring, and gating during DIBH treatment [9], [10], [11].

Despite these advantages, the current surface-guided DIBH workflow still typically requires two CT acquisitions: a DIBH-CT for treatment planning and a separate free-breathing CT (FB-CT) to provide a body contour for baseline surface registration during treatment setup at the stage of treatment [12], [13], [14], [15]. This dual acquisition increases imaging dose and adds time and complexity to the simulation process, which may create workflow inefficiencies in high-throughput clinics. In our clinic, routine biplanar CT scout images, specifically coronal and sagittal projections, are acquired at FB during CT simulation to localize the scan range. Although these scout images are two-dimensional (2D) projections, together they may contain sufficient information to approximate the patient's three-dimensional (3D) body contour. A recent study demonstrated the feasibility of generating 3D CT images directly from biplanar X-ray images [16]. Although prior studies have investigated three-dimensional anatomical or CT-like reconstruction from sparse projection data, including biplanar imaging, the use of routine biplanar CT scout images to generate a free-breathing body contour for baseline surface registration in surface-guided DIBH radiotherapy has not been established [17], [18], [19]. The specific clinical objective in this setting is not full CT intensity reconstruction, but generation of a geometrically accurate body contour suitable for patient setup and baseline surface registration for SGRT-guided DIBH radiotherapy. If a reliable three-dimensional free-breathing body contour could be derived directly from these routinely acquired scout images, this approach could reduce reliance on an additional free-breathing CT acquisition in selected workflows where free-breathing CT is primarily used to generate a baseline surface for surface-guided DIBH setup.

In this feasibility study, we investigated whether a patient-specific three-dimensional free-breathing body contour could be generated directly from routine biplanar CT scout images. Geometric agreement between the predicted and reference CT-based body contours was evaluated at both patient-wise and slice-wise levels. In addition, body-shape characteristics associated with model performance were assessed.

## 2 Material and methods

### 2.1 Clinical workflow overview

In the conventional surface-guided DIBH radiotherapy workflow, a pair of CT scout images is first acquired to verify patient positioning and define the scan range. Two helical CT scans are then obtained during treatment planning: a free-breathing CT (FB-CT) and a DIBH-CT. RT Structure Sets (RSs) are generated from both scans and are required for both setup and planning purposes. The FB-CT-derived RS provides the body contour for baseline surface registration, whereas the DIBH-CT-derived RS is used together with the DICOM RT Plan (RP) for treatment planning and beam delivery. During treatment, the FB-derived body contour is mainly used for initial rough setup, baseline surface registration, and verification of the free-breathing level. Subsequent fine positioning is performed using the DIBH reference surface, followed by image-guided verification such as cone-beam CT according to institutional practice. The DIBH-derived RS/RP is then used for treatment execution under breath-hold conditions.

**Fig. 1:**
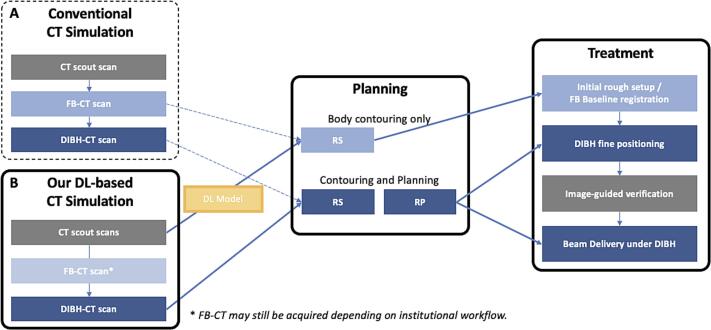
Conventional and proposed workflows for surface-guided DIBH radiotherapy. (A) In the conventional workflow, CT scout scans are followed by FB-CT and DIBH-CT acquisitions. The FB-CT-derived body contour is used for initial rough setup and FB baseline registration, whereas the DIBH-CT-derived RS/RP is used for treatment planning, DIBH fine positioning, image-guided verification, and beam delivery. (B) In the proposed workflow, biplanar CT scout images are used as input to the DL model to generate a predicted FB body contour and setup RS, while the DIBH-CT-based planning and treatment workflow remains unchanged. FB-CT may still be acquired for backup planning or other purposes depending on institutional workflow. Abbreviations: RS = DICOM RT Structure Set; RP = DICOM RT Plan; SGRT = surface-guided radiation therapy; FB = free breathing; DIBH = deep inspiration breath hold.

In the proposed workflow, a deep-learning (DL) model was developed to generate a 3D body mask from coronal and sagittal scout images, from which an RS could be generated for setup and baseline surface registration. The DIBH-CT-based planning and treatment delivery workflow remained unchanged. Fig. 1 summarizes the conventional and proposed workflows.

### 2.2 Study design and patient cohort

We retrospectively collected 173 thoracic CT-simulation studies performed on a Philips Brilliance Big Bore CT simulator (Philips Medical Systems, Eindhoven, the Netherlands) between March and May 2025. The cohort consisted of patients who underwent chest CT simulation during this period and was not restricted to a specific indication. This study was approved by the Institutional Review Board (IRB No. 2411–099-1587) and conducted in accordance with institutional privacy and research governance policies. For each study, we collected (i) paired coronal and sagittal CT scout images used to define the scan range and (ii) the corresponding free-breathing CT (FB-CT) image acquired under routine chest simulation conditions. All records were de-identified prior to analysis. The cohort included 173 patients (34 male and 139 female) with a median age of 57 years (range, 20–86 years). Patient demographics and imaging characteristics are summarized in Table 1.

**Table 1:** Patient cohort and imaging characteristics.

**Characteristics**	**Values**
Total studies (n)	173
Sex (M/F)	34 / 139
Age (years)	Median 57 (range 20–86)
CT simulator	Philips Brilliance Big Bore
CT scout protocol	120 kVp; 50 mA; mAs (not recorded); CTDI_vol_ 0.16 mGy
FB-CT protocol	120 kVp; 256.6 ± 95.4 mA; 157.8 ± 58.7 mAs; CTDI_vol_ 9.3 ± 3.5 mGy
CT scout image matrix and pixel size	512 × 512; 1.37 × 1.37 mm^2^
FB-CT image matrix, pixel size and slice thickness	512 × 512; 1.37 × 1.37 mm^2^ in 96 studies, 0.97–1.27 mm^2^ in the remaining studies; 3 mm or 5 mm

### 2.3 Data preprocessing

**Fig. 2:**
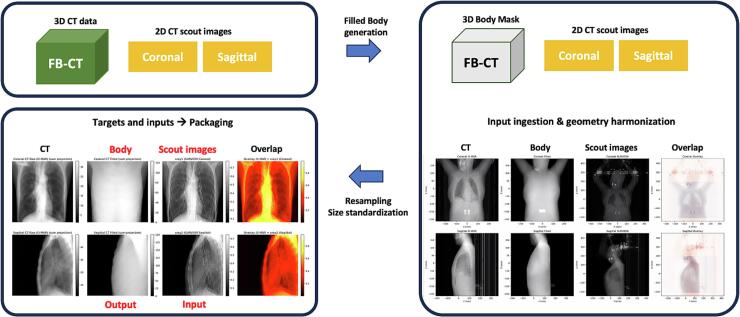
Data processing and packaging pipeline. From each CT-simulation session, the FB-CT and biplanar CT scout images (coronal, sagittal) are ingested and geometrically harmonized. The body (“filled-body”) mask is generated on the FB-CT, and inputs/targets are cropped in fixed millimeters, resampled to 1-mm^3^, and size-standardized (3D images and 2D scouts), with sum-projection and overlap views shown for quality check before packaging.

We implemented an automated preprocessing pipeline to convert each study into standardized input-output pairs for model development and evaluation. Fig. 2 summarizes the preprocessing workflow.

**Data arrangement:** For each CT-simulation study, paired coronal and sagittal CT scout images were matched with the corresponding FB-CT and RS (i.e., body contour). Nine studies with missing or inconsistent data components were excluded.

**Reference contour generation (3D body mask):** The FB-CT-derived body mask was generated using an automated threshold- and morphology-based procedure. An initial foreground mask was created on the FB-CT using CT > −1024 HU to identify the scan-region foreground and exclude voxels assigned to the background fill value. The mask was then refined using binary dilation with two iterations and axial slice-wise hole filling, repeated twice, followed by binary erosion with two iterations. Gaussian smoothing was applied with a sigma of 1.0 to reduce boundary irregularities, and the final binary body mask (inside = 1, outside = 0) was obtained using a threshold of 0.5 on the smoothed mask. The same thresholding and morphological-processing parameters were applied consistently to all studies. This final mask served as the reference contour and output label for model training and evaluation

**Data harmonization:** A thoracic field of view was defined to crop the scout images and the corresponding 3D body mask. The CT scout images and FB-CT image volumes were resampled to ensure consistent spacing, slice thickness, orientation, and image-space alignment. The scout images were resized to a unified in-plane resolution, and the cropped FB-CT image volume was resampled to 1 mm^3^ isotropic voxels, center-cropped to approximately 280 × 280 × 280 mm^3^, and then downsampled to a matrix size of 192 × 192 × 192 with a voxel size of 1 × 1 × 1 mm^3^. A lower-resolution setting of 128 × 128 × 128 voxels was used for ablation experiments.

**Input and output data:** The coronal and sagittal 2D scout images were used as inputs, and the corresponding 3D body masks were used as output labels.

### 2.4 Model architecture and training

We modified the X2CT architecture [16], a multi-view 2D-to-3D generative framework that translates biplanar 2D X-ray images to 3D CT images. X2CT was selected because it is directly compatible with paired coronal and sagittal projection inputs, allowing task-focused adaptation to body-contour prediction rather than full CT intensity reconstruction. The generator consisted of a multi-view 3D network with instance normalization, ReLU activation, no dropout, 64 base channels, and four down-sampling stages. Adversarial learning was implemented using a 3D discriminator together with multi-2D map discriminators operating on learned projections to encourage view-consistent reconstruction. Additional architectural details are described in the original X2CT paper [16]. Input voxel values were normalized to the range of 0–1. The network output was a continuous 3D image, and the final 3D body mask was obtained by applying a threshold of 0.5 after inference. Model training used the Adam optimizer (β₁ = 0.5, β₂ = 0.99) with an initial learning rate of 2 × 10^−4^ and a batch size of 2. The dataset was split at the patient level into training, validation, and test cohorts in a 70:15:15 ratio (121/26/26 studies). Training was performed for up to 200 epochs, consisting of 100 epochs at the initial learning rate followed by 100 epochs of linear decay. Model checkpoints were saved every 5 epochs, and the checkpoint with the lowest validation loss among the saved checkpoints (epoch 130) was selected for final evaluation on the held-out test cohort. Training and validation loss curves used for model selection are provided in Supplementary Fig. S1. The model was implemented in Python 3.12.7 and trained on an NVIDIA RTX A6000 GPU (48 GB) with CUDA 11.7.

### 2.5 Evaluation

Geometric agreement between the predicted body contour and the reference three-dimensional body mask was evaluated using the Dice coefficient (DC), 95th percentile Hausdorff distance (HD95; mm), and mean surface distance (MSD; mm). DC quantified global volumetric overlap, whereas HD95 and MSD assessed boundary agreement because DC may be relatively insensitive to localized surface deviations in large structures. HD95 represented large but non-extreme boundary deviations relevant to setup accuracy, and MSD quantified average surface discrepancy. Higher DC and lower HD95/MSD indicated better performance. Metrics were calculated in Python using the medpy.metric.binary module: dc() for DC, hd95() for HD95, and asd() for average surface distance, reported as MSD. Voxel spacing was specified using the voxelspacing argument.

Performance was assessed at patient-wise and slice-wise levels. Patient-wise DC, HD95, and MSD were computed for each study and summarized as mean ± standard deviation across the test cohort. Slice-wise metrics were calculated on individual axial slices to evaluate spatial variation along the superior-inferior direction.

To provide a clinically relevant surface-based assessment for free-breathing surface-guided setup, an anterior/anterior-lateral surface analysis was performed in the test cohort. For each axial slice, the anterior region was defined using the mid-coronal line of the reference body mask, and anterior-side surface points were selected. Surface agreement was evaluated using anterior-surface MSD, anterior-surface HD95, and the percentage of anterior surface points within 2, 3, and 5 mm of the reference surface.

An association analysis assessed whether body-shape characteristics were related to model performance. Four projection-based features were analyzed: mean coronal width, mean sagittal depth, left-right asymmetry ratio, and width coefficient of variation. These represented left-right body size, anterior-posterior body size, coronal asymmetry, and slice-to-slice width irregularity, respectively. Detailed definitions are provided in the Supplementary Methods. Spearman correlation analysis among DC, HD95, and MSD was also performed as a supplementary analysis.

For qualitative evaluation, cases with the highest and lowest patient-wise DC were identified as candidate best- and worst-performing cases. Their HD95 and MSD values were reviewed to confirm favorable and unfavorable surface-distance performance. Scout-image inputs, predicted contours, and reference contours were visually compared, and selected body-shape features were examined relative to the cohort distribution using z-score–based comparison.

## 3 Results

**Fig. 3:**
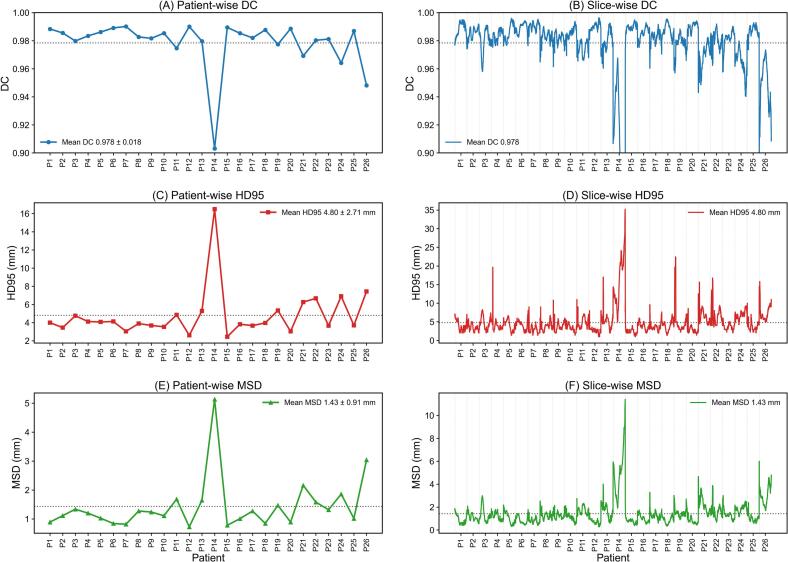
Patient-wise and slice-wise geometric performance in the independent test cohort. (A, C, E) Patient-wise Dice coefficient (DC), 95th percentile Hausdorff distance (HD95), and mean surface distance (MSD) for the 26 patients in the test cohort. (B, D, F) Slice-wise DC, HD95, and MSD across axial slices. DC, HD95, and MSD are shown in blue, red, and green, respectively. Horizontal gray dotted lines indicate mean values, and vertical dashed lines in the slice-wise plots indicate patient boundaries. (For interpretation of the references to colour in this figure legend, the reader is referred to the web version of this article.)

In patient-wise analysis, the mean Dice coefficient (DC) was 0.979 ± 0.017, the mean 95th percentile Hausdorff distance (HD95) was 4.23 ± 2.87 mm, and the mean surface distance (MSD) was 1.05 ± 0.73 mm. These findings indicate that the predicted contours closely matched the reference 3D body masks at both volumetric and surface levels. Fig. 3 summarizes both patient-wise and slice-wise geometric performance in the independent test cohort. Patient-wise distributions of DC, HD95, and MSD showed consistently favorable performance across most test patients, although an outlier case (P14) was observed. Slice-wise analysis similarly showed high overlap and low surface discrepancy across most axial slices, indicating that geometric agreement was generally preserved along the superior-inferior direction. Larger slice-wise deviations were mainly observed in P14, suggesting reduced stability in a patient with more challenging body geometry. Patient-wise slice distributions of DC, HD95, and MSD are further shown in Supplementary Fig. S2.

In the additional anterior/anterior-lateral surface analysis, the model achieved an anterior-surface MSD of 1.46 ± 1.30 mm and an anterior-surface HD95 of 4.80 ± 3.36 mm in the test cohort. The percentages of anterior surface points within 2, 3, and 5 mm were 76.92 ± 14.84%, 86.30 ± 13.91%, and 94.47 ± 10.88%, respectively. These results provide clinically interpretable surface-distance measures for the surface region most relevant to FB SGRT setup. Detailed patient-wise results are provided in Supplementary Fig. S3 and Table S1.

**Fig. 4:**
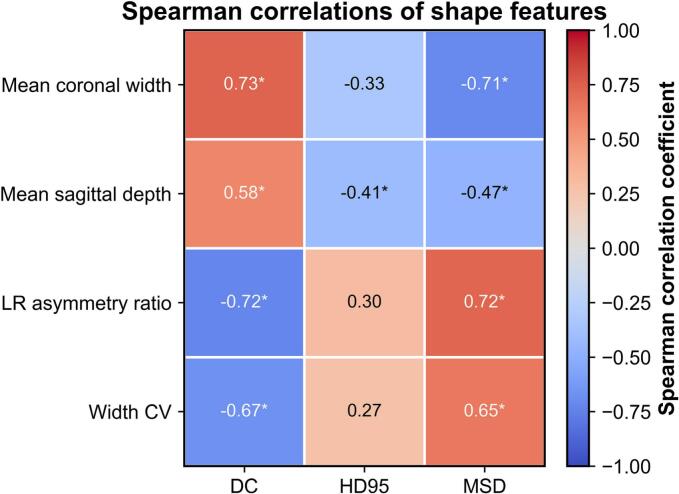
Spearman correlations between body-shape features and geometric performance metrics. Heatmap showing Spearman correlation coefficients between four body-shape features and patient-wise geometric performance metrics in the independent test cohort. Asterisks indicate statistically significant associations (*p* < 0.05).

To further investigate whether model performance varied according to patient body-shape characteristics, correlation analysis was performed between selected body-shape features and prediction performance. Detailed definitions and calculation procedures are provided in the Supplementary Methods. Overall, the correlation analysis showed that model performance was associated with patient body-shape characteristics. Larger body-size features, including mean coronal width and mean sagittal depth, tended to be associated with better performance, whereas greater left-right asymmetry and slice-to-slice width variation tended to be associated with poorer performance. Representative associations included a positive correlation between DC and mean coronal width (ρ = 0.73, *p* < 0.05) and a negative correlation between DC and left-right asymmetry ratio (ρ = −0.72, p < 0.05). These findings suggest that the model performed more accurately in patients with larger, more symmetric, and geometrically regular torso shapes. Fig. 4 summarizes the full set of Spearman correlations, and the corresponding *p*-values are provided in Supplementary Table S2.

**Fig. 5:**
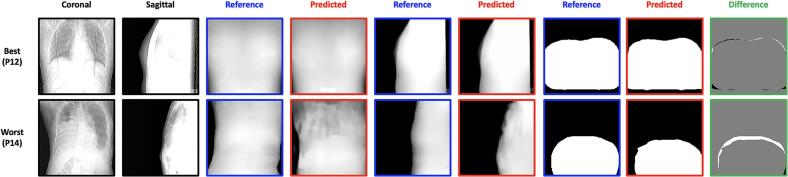
Representative best- and worst-performing cases. Input scout images, reference contours, predicted contours, and transaxial mid-slice comparison for the best-performing case (P12) and worst-performing case (P14).

As described above the representative cases were those with the highest and lowest DC values. Fig. 5 shows the qualitative comparison of scout-image inputs, predicted contours, and reference contours. The observed discrepancy in the lowest-DC case did not appear to be primarily explained by obvious projection mismatch, and may instead reflect limited model generalization to less-represented body-shape patterns. The corresponding body-shape feature distributions and absolute z-scores are provided in Supplementary Fig. S5 and Supplementary Table S3, respectively. The best-performing case showed a relatively large, symmetric, and geometrically regular body shape, whereas the worst-performing case showed a narrower body envelope, greater asymmetry, and larger shape irregularity relative to the overall cohort distribution.

## 4 Discussion

This feasibility study suggests that routinely acquired biplanar CT scout images contain sufficient geometric information to generate a three-dimensional free-breathing body contour using deep learning. In the retrospective test cohort, the model showed high agreement between predicted and reference body masks, with a mean Dice coefficient (DC) of 0.979 ± 0.017, 95th percentile Hausdorff distance (HD95) of 4.23 ± 2.87 mm, and mean surface distance (MSD) of 1.05 ± 0.73 mm. Slice-wise performance was generally stable across the thorax, although one subject was an outlier. Anterior/anterior-lateral surface analysis showed an MSD of 1.46 ± 1.30 mm and HD95 of 4.80 ± 3.36 mm, with 76.92 ± 14.84%, 86.30 ± 13.91%, and 94.47 ± 10.88% of anterior surface points within 2, 3, and 5 mm, respectively. These results complement the global metrics, although actual SGRT setup validation remains necessary.

In surface-guided DIBH practice, the FB body contour is used for baseline surface registration, whereas DIBH-CT is used for treatment planning. Predicting the FB body contour from routine scout images may reduce reliance on additional FB-CT in selected workflows, lowering imaging dose and simulation burden when FB-CT is acquired primarily for baseline surface generation. In our cohort, scanner-reported CTDIvol was 9.34 ± 3.48 mGy for FB-CT and 0.162 mGy for scout images. However, FB-CT may also serve as a backup planning dataset if a patient cannot maintain DIBH, and the proposed method should not be interpreted as a universal replacement. In our workflow, patients are screened for DIBH capability before CT simulation; nevertheless, prospective validation is required before modifying acquisition protocols. Because FB contours are mainly used for initial setup and baseline registration, whereas fine positioning uses the DIBH reference surface and image guidance, the predicted contour need not be geometrically perfect but should support reliable initial setup. Thus, the present findings demonstrate geometric feasibility rather than setup equivalence, and clinical validation remains necessary before adoption [20], [21], [22].

An important finding was that performance depended partly on body-shape characteristics. The model performed better in patients with larger, more symmetric, and more regular torso shapes, whereas asymmetry and irregularity were associated with reduced accuracy (Fig. 3, Fig. 4 and Supplementary Table S2). This should be interpreted cautiously because DC may be relatively insensitive to localized surface deviations in large structures. Similar but weaker trends were observed for HD95 and MSD, suggesting that the association between body size and performance may reflect both true prediction differences and metric behavior. Representative case analysis supported this interpretation: the best-performing case had a large, symmetric, and regular body shape, whereas the worst-performing case had a narrow, asymmetric, and irregular torso geometry (Fig. 5). Importantly, observed model failures were more likely attributable to limited generalization to less-represented body-shape patterns, supporting the interpretation that body-shape irregularity and asymmetry contributed to the outlier case (P14, Fig. 5 and Supplementary Fig. S5).

This study has several limitations. First, the model was developed and evaluated using a limited single-institution, single-scanner dataset, which may limit generalizability. Although the cohort was not restricted to a specific indication, its distribution reflected our institutional case mix. The model was also trained without stratification by sex, indication, or surgical status. Sex-related thoracic surface differences, particularly in the anterior chest and breast region, and breast or chest wall surgery may affect prediction performance. Because the cohort was sex-imbalanced and included few male patients, sex-specific training was not performed and should be evaluated in larger, balanced datasets. Second, the CT-derived body mask was treated as the reference standard, although it may be affected by thresholding and morphological processing. In addition, FB-CT may represent averaged anatomy over multiple breathing cycles, and respiratory-level differences between scout images and FB-CT may introduce geometric mismatch. Therefore, residual surface discrepancies of approximately 1 mm may reflect prediction error, reference-mask uncertainty, and respiratory variability. Third, SGRT setup accuracy, baseline registration, gating thresholds, and end-to-end delivery were not directly evaluated. Fourth, coordinate-level spatial fidelity within the DICOM patient coordinate system, including translational and rotational consistency and isocenter alignment, was not explicitly validated.

Future work should therefore include clinical validation using larger multi-center and multi-scanner datasets with diverse anatomical scenarios, as well as prospective patient studies integrated into actual SGRT-guided DIBH workflows. Further investigation is also needed to determine whether shape-dependent failure modes can be incorporated into practical acceptance criteria, automated quality-assurance procedures, and fail-safe strategies for deciding when a standard FB-CT should still be acquired.

## CRediT authorship contribution statement

**Yoonsuk Huh:** Writing – review & editing, Writing – original draft, Visualization, Validation, Supervision, Software, Resources, Project administration, Methodology, Investigation, Funding acquisition, Formal analysis, Data curation. **Seonghee Kang:** Writing – review & editing, Project administration, Investigation, Funding acquisition, Formal analysis, Data curation. **Jaewon Yang:** Writing – review & editing, Validation, Software, Methodology, Formal analysis. **Jung-in Kim:** Conceptualization, Methodology, Supervision, Project administration, Writing – review & editing.

## Declaration of competing interest

The authors declare that they have no known competing financial interests or personal relationships that could have appeared to influence the work reported in this paper.

## Appendix A Supplementary data

Supplementary methods and results, including model-selection details, definitions of body-shape features, supplementary statistical analyses, Supplementary Figures S1–S3, and Supplementary Table S1.
