# Supplementary material for: Feasibility of predicting free-breathing body contours from biplanar CT scout images for surface-guided DIBH radiotherapy
Source: Phys Imaging Radiat Oncol. 2026 Jun 17;40:101023. doi: 10.1016/j.phro.2026.101023 (PMC13324509; doi:10.1016/j.phro.2026.101023)
Supplement: Supplementary file 1 — Supplementary methods and results, including model-selection details, definitions of body-shape features, supplementary statistical analyses, Supplementary Figures S1–S3, and Supplementary Table S1. [file mmc1.docx]

**Supplementary Methods**

**S1. Model selection based on training and validation loss**

Training and validation losses were recorded throughout optimization to document model-selection behavior. Loss curves over 200 epochs are provided in Supplementary Figure S1. Because model checkpoints were saved every 5 epochs, the final model for test evaluation was selected as the saved checkpoint with the lowest validation loss. Among the saved checkpoints, the minimum validation loss was observed at epoch 130; this checkpoint was therefore used for final evaluation on the held-out test cohort.

**S2. Definition of body-shape features**

To explore whether patient body shape was associated with contour-prediction performance, four projection-based body-shape features were extracted from the three-dimensional binary filled-body mask. These features were selected to represent body size, symmetry, and slice-to-slice geometric regularity.

S2.1. Mean coronal width

Mean coronal width was defined as the average left-right body extent across axial levels. First, the three-dimensional body mask $M\left( z, y, x \right)$ was projected along the anterior-posterior direction to generate a coronal projection mask:

$$C\left( z, x \right)=1\left( \sum_{y} M\left( z, y, x \right)>0 \right)$$

For each axial level z, the coronal width was calculated as:

$$w_{cor}\left( z \right)=\sum_{x} C(z, x)$$

Mean coronal width was then computed as:

$$mean\_cor\_width=\frac{1}{N_{z}}\sum_{z} w_{cor}(z)$$

This feature represents the average left-right body size in the coronal view. Larger values indicate a wider torso silhouette.

S2.2. Mean sagittal depth

Mean sagittal depth was defined as the average anterior-posterior body extent across axial levels. The body mask was projected along the left-right direction to generate a sagittal projection mask:

$$S\left( z, y \right)=1\left( \sum_{x} M\left( z, y, x \right)>0 \right)$$

For each axial level z, the sagittal depth was calculated as:

$$d_{sag}\left( z \right)=\sum_{y} S(z, y)$$

Mean sagittal depth was then computed as:

$$mean\_sag\_depth=\frac{1}{N_{z}}\sum_{z} d_{sag}(z)$$

This feature represents the average anterior-posterior body size in the sagittal view. Larger values indicate a deeper or thicker torso silhouette.

S2.3. Left-right asymmetry ratio

Left-right asymmetry ratio was used to quantify body-shape asymmetry in the coronal projection. The coronal projection mask $C(z, x)$ was divided at the midline into left and right halves. Their areas were calculated as:

$$A_{L}=\sum_{z} \sum_{x<x_{mid}} C(z, x)$$

$$A_{R}=\sum_{z} \sum_{x\geq x_{mid}} C(z, x)$$

The asymmetry ratio was then defined as:

$$lr\_asym\_ratio=\frac{\left| A_{L}-A_{R} \right|}{A_{L}+A_{R}}$$

Values close to 0 indicate a more symmetric body shape, whereas larger values indicate greater left-right asymmetry.

S2.4. Width coefficient of variation

Width coefficient of variation (width CV) was used to quantify slice-to-slice irregularity of body width along the superior-inferior axis. Using the coronal width values $w_{cor}\left( z \right)$, the mean and standard deviation were calculated as:

$$\mu_{w}=\frac{1}{N_{z}}\sum_{z} w_{cor}(z)$$

$$\sigma_{w}=\sqrt{\frac{1}{N_{z}-1}\sum_{z} \left( w_{cor}\left( z \right)-\mu_{w} \right)^{2}}$$

The width coefficient of variation was then defined as:

$$witdh\_cv=\frac{\sigma_{w}}{\mu_{w}}$$

Lower values indicate a smoother and more uniform body shape along the superior-inferior direction, whereas larger values indicate greater slice-to-slice variation and increased geometric irregularity.

**S3. Supplementary statistical analyses**

For exploratory analysis, Spearman correlation coefficients were calculated between the four body-shape features and patient-wise geometric performance metrics (DC, HD95, and MSD). In addition, best- and worst-performing cases were compared with the overall cohort distribution using z-scores, defined as:

$$z=\frac{x-\mu}{\sigma}$$

where $x$ is the feature value for a given patient, $\mu$ is the cohort mean, and $\sigma$ is the cohort standard deviation.

**Supplementary Results**

**S1. Training and validation loss curves**

Supplementary Figure S1 shows the training and validation loss curves over 200 epochs. Both losses decreased during training and then gradually plateaued. Among the checkpoints saved every 5 epochs, the lowest validation loss was observed at epoch 130, which was therefore selected for final evaluation on the held-out test cohort.


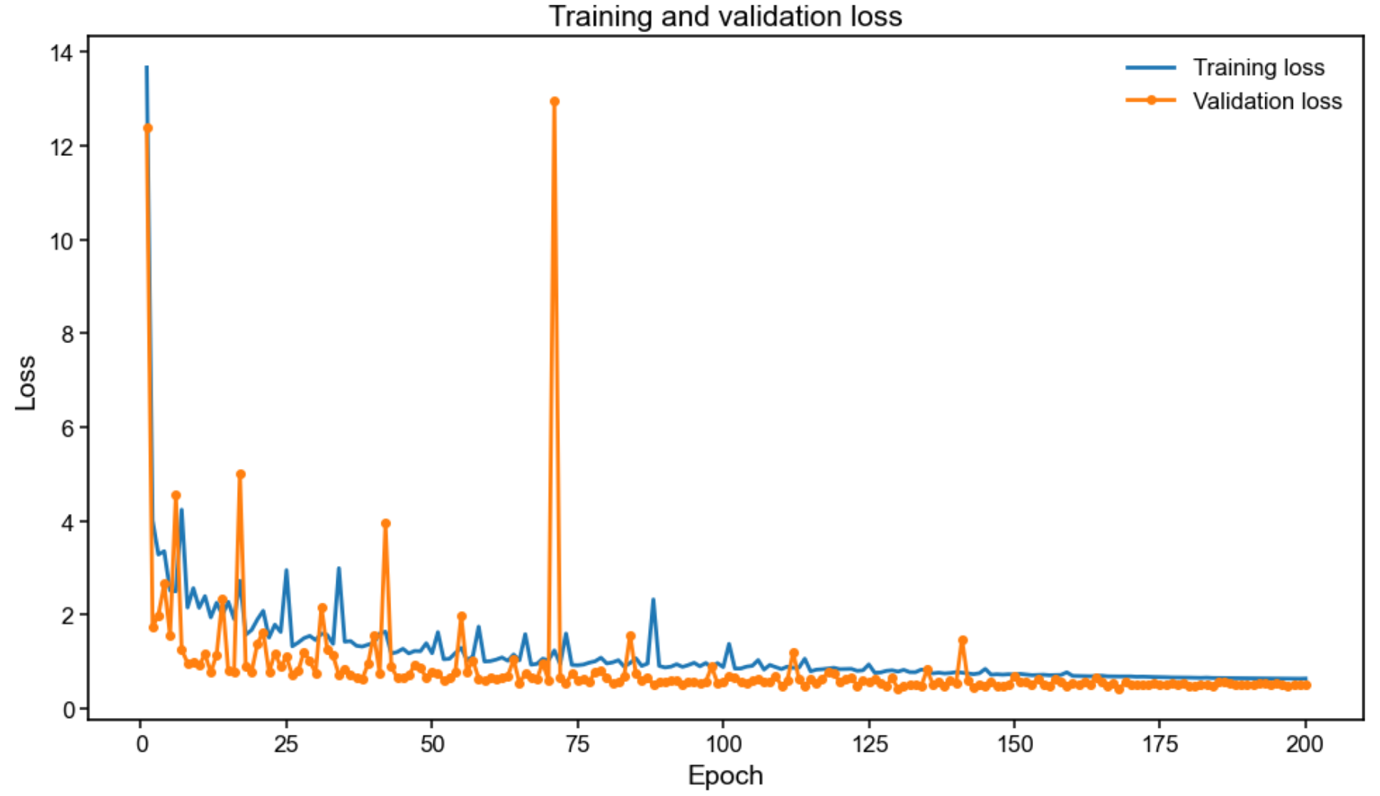


**Supplementary Figure S1**. Training and validation loss curves over 200 epochs. Checkpoints were saved every 5 epochs. The checkpoint at epoch 130 showed the lowest validation loss among the saved checkpoints and was selected for final evaluation on the held-out test cohort.

**S2. Patient-wise slice distributions of geometric performance metrics**

To further examine within-patient variability, slice-wise distributions of DC, HD95, and MSD were summarized for each patient in the independent test cohort using box plots. Most patients showed tightly clustered slice-wise DC values near 1.0, with correspondingly low HD95 and MSD values, indicating stable geometric agreement across the superior-inferior extent. However, a subset of patients demonstrated broader slice-wise distributions and more frequent outliers, suggesting increased spatial variability in prediction performance within those cases. In poorer-performing cases, the reduced agreement appeared to extend across multiple slices rather than being limited to only a few isolated outliers.


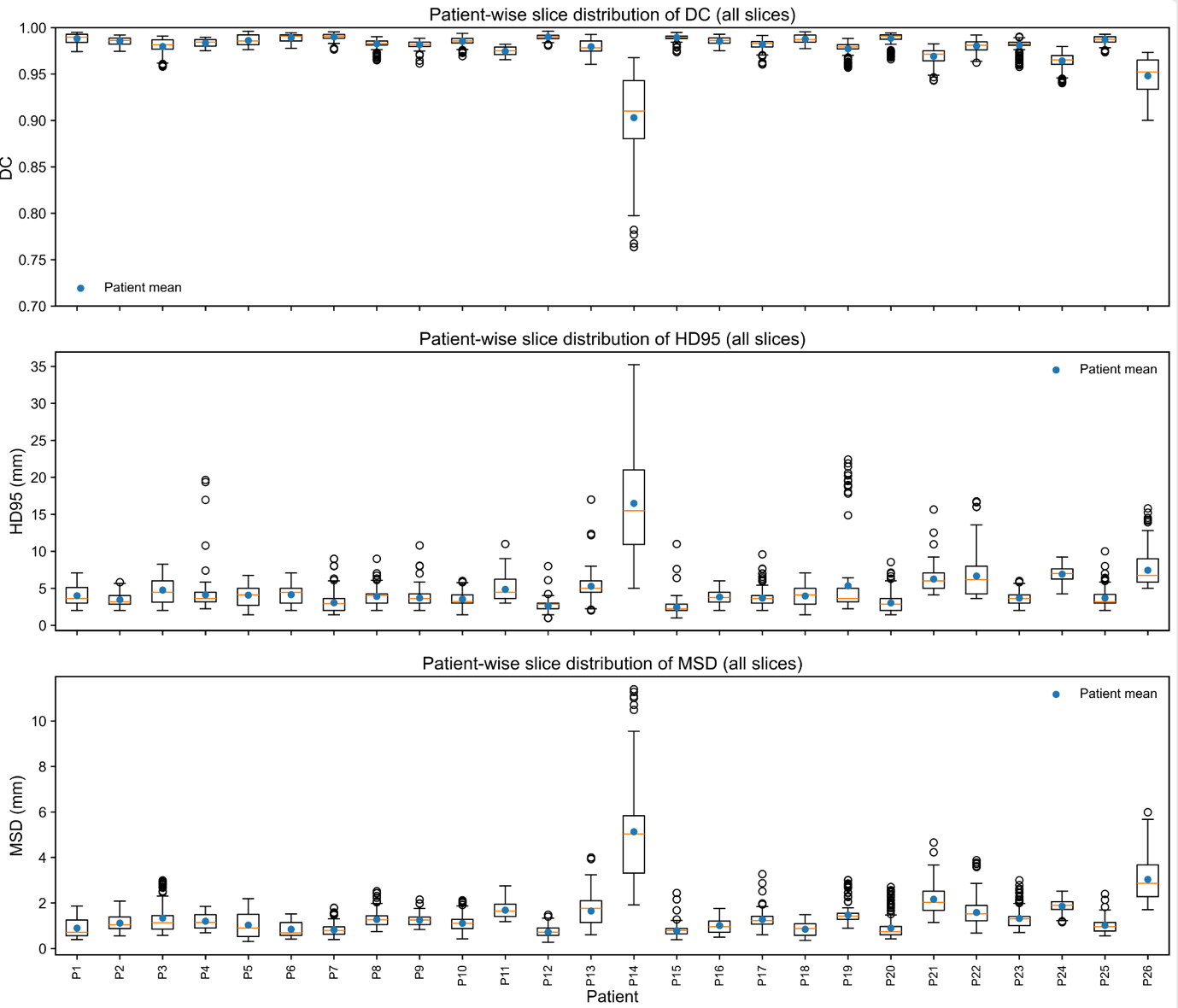


**Supplementary Figure S2.** Patient-wise slice distributions of geometric performance metrics in the independent test cohort. Blue dots indicate patient-wise mean values across slices.

**S3. Correlations among patient-wise geometric performance metrics**

Supplementary Figure S3 shows the pairwise Spearman correlations among patient-wise DC, HD95, and MSD. DC was inversely correlated with HD95 and MSD, where as HD95 and MSD were positively correlated.


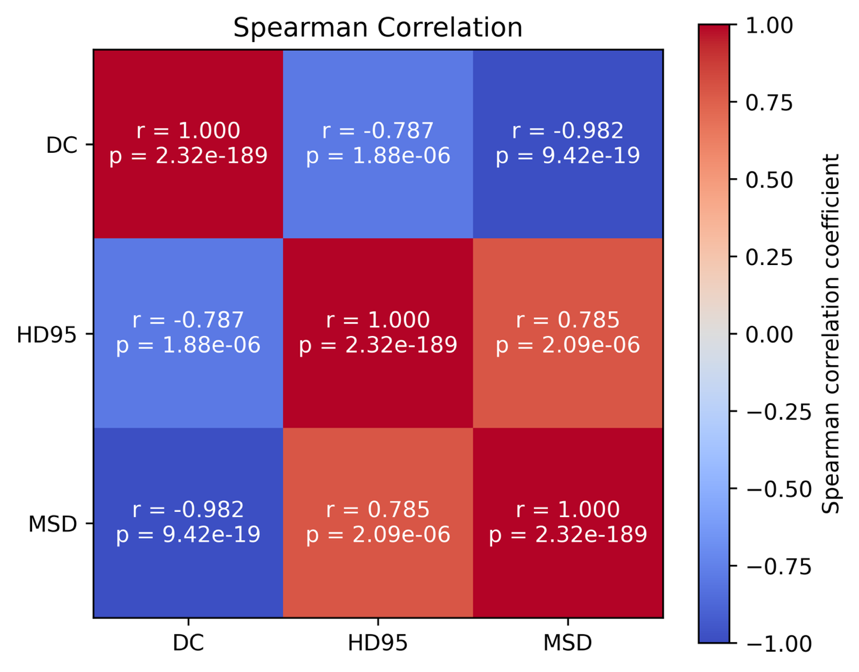


**Supplementary Figure S3.** Pairwise Spearman correlation heatmap of mean DC, HD95, and MSD across the independent test cohort. Values in each cell indicate the correlation coefficient (r) and corresponding p-value.

**S4. P-values for correlations between body-shape features and performance metrics**

Supplementary Table S1 summarizes the p-values for Spearman correlations between the four selected body-shape features and patient-wise geometric performance metrics. Significant associations were observed primarily for DC and MSD, whereas associations with HD95 were generally weaker.

**Supplementary Table S1.** P-values for Spearman correlations between body-shape features and patient-wise performance metrics.

|  | DC | HD95 | MSD |
| --- | --- | --- | --- |
| Mean coronal width | <0.001 | 0.1038 | <0.001 |
| Mean sagittal depth | 0.0016 | 0.0368 | 0.0158 |
| LR asymmetry ratio | <0.001 | 0.1309 | <0.001 |
| Width CV | <0.001 | 0.1794 | <0.001 |
